# Post-prandial carbohydrate ingestion during 1-h of moderate-intensity, intermittent cycling does not improve mood, perceived exertion, or subsequent power output in recreationally-active exercisers

**DOI:** 10.1186/1550-2783-10-4

**Published:** 2013-01-24

**Authors:** Eric K O’Neal, Sylvia P Poulos, Jonathan E Wingo, Mark T Richardson, Phillip A Bishop

**Affiliations:** 1 Department of Kinesiology, The University of Alabama, Tuscaloosa, AL, USA; 2 Global Research, The Coca-Cola Company, Atlanta, GA, 30313, USA; 3 Current address: Department of Health, Physical Education, and Recreation, University of North Alabama, Florence, AL, 35632, USA

**Keywords:** Recreational exercise, Non-caloric, Sport beverage, RPE, POMS, Pre-exercise meal

## Abstract

**Background:**

This study compared the effects of ingesting water (W), a flavored carbohydrate-electrolyte (CE) or a flavored non-caloric electrolyte (NCE) beverage on mood, ratings of perceived exertion (RPE), and sprint power during cycling in recreational exercisers.

**Methods:**

Men (*n* = 23) and women (*n* = 13) consumed a 24–h standardized diet and reported 2–4 h post-prandial for all test sessions. After a familiarization session, participants completed 50 min of stationary cycling in a warm environment (wet bulb globe temperature = 25.0°C) at ~ 60-65% of heart rate reserve (146 ± 4 bpm) interspersed with 5 rest periods of 2 min each. During exercise, participants consumed W, CE, or NCE, served in a counterbalanced cross-over design. Beverage volume was served in 3 aliquots equaling each individual’s sweat losses (mean 847 ± 368 mL) during the familiarization session. Profiles of Mood States questionnaires (POMS) were administered and blood glucose levels were determined pre- and post- sub-maximal cycling. Following sub-maximal exercise, participants completed 3 30–s Wingate anaerobic tests (W_AnT_) with 2.5 min rest between tests to assess performance.

**Results:**

Blood glucose was higher (p <  0.05) after 50 min of submaximal cycling just prior to the W_AnT_ for CE (6.1 ± 1.7 mmol/L) compared to W (4.9 ± 1.5 mmol/L) and NCE (4.6 ± 1.2 mmol/L). Nonetheless, there were no differences among treatments in peak (642 ± 153, 635 ± 143, 650 ± 141 watts for W, NCE, and CE, respectively; p  =  0.44) or mean (455 ± 100, 458 ± 95, 454 ± 95 watts for W, NCE, and CE, respectively; p = 0.62) power for the first W_AnT_ or mean (414 ± 92, 425 ± 85, 423 ± 82 watts, respectively; p = 0.13) power output averaged across all 3 W_AnT_. Likewise, RPE during submaximal exercise, session RPE, and fatigue and vigor assessed by POMS did not differ among beverage treatments (p > 0.05).

**Conclusions:**

Carbohydrate ingestion consumed by recreational exercisers during a 1–h, moderate-intensity aerobic workout did not alter mood or perceived exertion, nor did it affect subsequent anaerobic performance under the conditions of this study. Drinking caloric sport beverages does not benefit recreational exercisers in a non-fasted state.

## Background

Multiple investigations have found ingestion of carbohydrate-electrolyte beverages (CE) before or during exercise improves performance during high-intensity, continuous endurance exercise lasting 1 h or less [1-11] and during intermittent, high-intensity exercise simulating sports such as soccer or basketball lasting approximately 1 h [12,13]. The mechanisms for performance improvements related to CE consumption during shorter-bout activity are not well understood. Multiple investigations in which rinsing a carbohydrate-containing solution in the mouth without ingestion improves performance lasting 1 h or less [14-18] has led to a hypothesis suggesting that performance enhancement may be linked to centrally mediated factors involving receptors in the oral cavity associated with reward and locomotion centers that are activated when carbohydrates are sensed in the mouth [16].

Additional evidence suggesting decreased perceived exertion and alterations in mood from CE use can also be found. Backhouse et al. [19] found that cyclists reported higher levels of pleasure beginning at 15 min and persisting during a 2-h ride when consuming a CE versus an artificially sweetened placebo. Similarly, Rollo et al. [15] found runners reported greater feelings of pleasure in the first 5 minutes of a 30-min run at a self-selected pace with a CE mouth rinse versus a placebo. Additionally, in two studies [12,13] in which participants consumed CE during intermittent high intensity exercise for 1 h tended to report less fatigue and more vigor late in exercise compared to artificially sweetened placebos. Lower rate of perceived exertion (RPE) was also noted when cyclists consumed a carbohydrate beverage versus placebo during a 50 min of high intensity cycling followed by a Wingate Anaerobic Test [5].

The reputed benefits of CE ingestion described above may be related, in part, to study design. In a review of the literature concerning the efficacy of commercially available CE, Coombes and Hamilton [20] noted that studies supporting the use of CE for improved performance during prolonged endurance exercise frequently included participants exercising after a 12-h fast. Similar conditions were found for the majority of the ~ 1–h duration studies cited above in which positive results were found for carbohydrate beverages [2,4-9,11-15,17]. Of the 17 studies reviewed in this current paper, five [3,6,10,16,18] reported a benefit of CE use for subjects who were not fasted prior to exercise, and 1 of those investigations only included 5 moderately trained participants [10]. Cyclists [21] and runners [22] who were fed before exercise failed to show improved performance during 1-h time trials when consuming CE as compared to a sweetened placebo during exercise. Ingesting carbohydrate-rich gels with water before and during runs lasting 75 min also has also not proven effective in improving performance of fed runners [23]. Similarly, the ergogenic effect of a carbohydrate mouth rinse reported in the studies mentioned above has not been confirmed in fed runners [24] or cyclists [25]. Conflicting results and few investigations in which a pre-exercise meal was consumed make it difficult to extrapolate results to individuals who are fed prior to exercise.

Given the preceding discussion, it remains unknown whether CE improves performance in recreational exercise bouts lasting ~ 1 h. Non-caloric electrolyte beverages (NCE), similar to the placebos prepared and used in the investigations cited above, may be an appealing alternative to water for exercisers concerned with caloric intake but who prefer flavored beverages over water, potentially increasing fluid intake during and after exercise [26]. However, it is unclear whether a NCE is as efficacious as a CE in improving or maintaining performance in recreational exercise bouts lasting ~ 1 h. Therefore, the purpose of this study was to determine if recreational exercisers, while in a post-prandial state, would; a) exhibit improved performance in exercise lasting ~ 1 h in duration, b) perceive exercise as less difficult, or c) report lower levels of fatigue, when consuming a CE during exercise compared to a NCE or water (W). It was hypothesized that there would be no differences in performance, mood, or rate of perceived exertion among beverages.

## Methods

### Participants

Men (n = 23) and women (n = 13) ages 19–30 who reported participating in a minimum of 150 but no more than 450 min of aerobic exercise per week for the previous 3 months volunteered to participate in this study. Thirteen of the thirty-six participants reported that they engaged in indoor or outdoor cycling (2.3 ± 1.4 times per week). Three participants reported engaging in organized endurance competitions in the last 6 months and none of the reported finishing times would classify these participants as elite level endurance athletes. During their initial familiarization session, participants completed a questionnaire concerning their current use of sport beverages. Anthropometric data and reported exercise frequency and duration are listed in Table 1. The study was approved by, and conducted in accordance with, guidelines of the University of Alabama’s Institutional Review Board, and participants provided written informed consent prior to beginning any study procedures.

**Table 1:** Characteristics of participants

	**Men*(n = *23)**	**Women*(n = *13)**	**Total*(n = *36)**
Age (years)	23 ± 3	24 ± 3	23 ± 3
Height (cm)	177 ± 7	165 ± 5	173 ± 9
Weight (kg)	77.6 ± 8.9	60.5 ± 9.1	71.4 ± 12.1
Body Mass Index (kg/m^2^)	24.6 ± 2.2	22.3 ± 2.9	23.8 ± 2.7
Body Fat (%)	10.3 ± 4.8	18.2 ± 4.6	13.2 ± 6.0
Aerobic Exercise Sessions (per week)	3.8 ± 1.1	4.5 ± 1.0	4.1 ± 1.1
Average Exercise Session Duration (minutes)	48.2 ± 20.5	57.3 ± 19.0	51.6 ± 20.1

Data are mean  ±  SD.

### Familiarization session

Prior to beginning experimental trials, participants completed a familiarization session designed to acquaint them with the exercise protocols and subjective rating scales. This session also permitted the estimation of total 1-h exercise sweat loss, as determined by body weight change, which was used to control fluid intake in all subsequent treatment sessions.

Participants were instructed to drink ~500 mL of water between their last meal and the time they went to bed the night before testing and a second 500 mL of water during the 2 hours before reporting to the laboratory. They were also instructed to avoid alcohol and caffeine during the 24-h period prior to experimental trials and to arrive at the laboratory at least 2 hours after eating. Upon arrival, body weight while wearing shorts, a t-shirt, and undergarments was measured using a beam-balance scale. Height was measured using a stadiometer integrated with the scale (Detecto, Webb City, MO) and body mass index (kg/m^2^) was recorded. The sum of skinfolds from three sites (Lange Caliper, Beta Technology Inc., Deer Park, NY) were recorded in accordance with American College of Sports Medicine guidelines [27] and used to estimate body fat percentage [28].

Heart rate (HR) was recorded (Team System Monitor, Polar Electro Oy, Kempele, Finland) continuously in 5-s intervals while subjects sat quietly for 15 min in a dimly lit room. The average HR from min 5 to 15 was determined. At the end of the 15– min rest period, a capillary blood sample was collected using the finger prick method. Whole blood was collected in a 100–μL fluoride/heparin/nitrite-containing capillary tube and mixed for 3 min before being analyzed in triplicate (PGM7 Analyzer, Analox Instruments, Lunenburg, MA) to confirm participants exhibited a normal blood glucose profile. The average of the 2 closest measurements was recorded.

A Profile of Mood States-Brief questionnaire (POMS) [29] was administered prior to exercise. Following the completion of the POMS, participants were fitted to ride a stationary bike (Johnny G’s Spinner Pro, Star Trac, Irvine, CA). Seat and handlebar height were recorded and were replicated for subsequent experimental trials. Participants spent 60 min in a heated environmental chamber (WBGT = 25.1 ± 0.3°C), completing 5 consecutive 10 min repetitions of of cycling interspersed by seated rest without pedaling for 2 min at minutes 10, 22, 34, 46 and 58 for a total of 50 min of cycling. Participants cycled at a HR corresponding to 60%-65% of HR reserve [30]. Rest periods were used to apply sweat patches and collect sweat for a separate investigation [31] during beverage treatment trials. The WBGT used in the test is equivalent to typical early morning and late evening summer conditions the participants would experience in the region in which they lived [26] and ensured adequate sweat rates required for an additional sweat profile study taking place.

The HR range chosen was intended to produce a moderate-intensity workout for a recreational exerciser. Participants self-selected the pedal cadence they wished to use and the resistance on the bike was gradually increased until they reached an intensity level that would allow them to maintain the target HR. Prescribed HR ranges were posted in front of the participants, and HR monitor displays provided each participant with visual and audible signals to assist with maintaining HR within the target range. After 5 min of cycling in the pre-determined HR range, participants were asked if they felt the intensity was below or above their normal exercise intensity. Intensity was adjusted between 5 to 10 beats per minute until it more closely matched their normal exercise intensity. Participants reported having no problem maintaining the prescribed intensity level. No fluid was consumed during the familiarization submaximal exercise bout. Immediately following the sub-maximal cycling bout a second POMS was administered and blood glucose was collected in a standardized 5-min period. Participants then completed a set of 3 Wingate Anaerobic Tests (W_AnT_) of 30–s duration with a resistance equal to ~ 7% of their body weight on an electronically-braked cycle ergometer (Velotron, RacerMate Inc., Seattle, WA). Participants continued pedaling at a resistance level and cadence of their choice during a 2.5 min recovery period after each W_AnT_.

Seat height adjustments were made to accommodate the subject and recorded for duplication during subsequent trials. Ratings of perceived exertion were measured using a 6 – 20 scale [32] at minutes 0, 20, 40 and 60. Upon completion of the W_AnT_ the participants changed back into their dry clothing and body weights were measured on the beam scale as before. Estimated sweat loss was determined from the change in pre- to post-exercise weight and accounted for voids when applicable. Session RPE (S-RPE) was reported ~15 min after the final W_AnT_.

### Experimental treatment sessions

Seven days after the familiarization session, participants returned to the laboratory to begin the first experimental trial. All tests for a given participant were on the same day of week. Participants were instructed not to change their current exercise routines and abstain from vigorous exercise 24 h before and on the day of testing. Participants were reminded to drink ~ 500 mL of water between their last meal and the time they went to bed the night before testing and a second 500 mL of water during the 2 hours before reporting to the laboratory. Participants were instructed to avoid alcohol and caffeine during the 24–h period prior to any experimental trial and to arrive at the laboratory at least 2 and no more than 4 hours after eating.

Trials were completed in a counterbalanced fashion, and treatment orders were randomly assigned to participants. Up to 3 participants cycled at the same time. Sessions for individuals took place at the same time of day, on the same day of the week, and with the same cohort of riders as the initial session. Testing took place over 4 weeks with 7 days separating trials excluding a few trials separated by 14 days because of scheduling conflicts.

During the 24-h period leading up to the first beverage consumption session, participants were provided with beverages, 3 meals and 2 snacks, which did not include meat products. Participants recorded the time each item was consumed and replicated consumption patterns for the following 2 sessions. Participants were required to consume all items they had been provided and no additional food or beverages (except for water) were permitted during the 24 h before testing. The last meal was eaten ~ 2–4 h prior to the commencement of exercise and only water was consumed in the 2–h period before reporting to the laboratory. Test sessions were held throughout the day, and the same pre-exercise meal was constant between trials for each individual. The total caloric value of the 24–h diet provided to participants was ~ 8,270 kJ containing 67.0% carbohydrate, 23.7% fat, and 9.3% protein based on the nutrition label on food item packaging.

As with the familiarization session, upon arrival, participants’ body weights were measured in minimal clothing. After participants changed into exercise attire, a POMS was administered and pre-exercise blood glucose was recorded. Participants then completed an exercise bout identical to that of the familiarization session in an environmental chamber maintained at a wet bulb globe temperature (WBGT) of approximately 25°C. Rate of perceived exertion was recorded at minutes 20, 40, and 60, and sweat patches were applied or sweat was collected from the participant’s lower back at minutes 12, 22, 34, 46, and 58 requiring the participants to stop cycling and remain seated on the stationary bike for 2 min resulting in 60 min of heat exposure and 50 total min of cycling as in the familiarization session. After the submaximal cycling bout was completed, subjects exited the chamber and sat in a chair for 5 min. Blood was collected via finger prick method for measurement of blood glucose and participants completed a second POMS questionnaire. Participants then mounted an electronically-braked cycle ergometer (Velotron, RacerMate Inc., Seattle, WA) and completed 3 Wingate Anaerobic Tests (W_AnT_) lasting 30 s each, and utilizing a resistance equal to ~7% body weight, with 2.5 min passive recovery between each test. Peak power and mean power were recorded for each W_AnT_. After each W_AnT_, participants continued pedaling at a resistance level and cadence of their choice for 2.5 min. During all W_AnT_, participants were given strong verbal encouragement. Following the third W_AnT,_ participants were given a short time (~15 min) to recover, towel off and have post-exercise weight measured before reporting their session-RPE. Additionally, a 2-item questionnaire was administered to assess the difficulty of the exercise session compared to participants’ normal workouts and to assess their beliefs regarding whether drinking the assigned beverage improved their performance ability. Each question was assessed using a 100-mm visual analog scale. The same investigator collected and recorded all glucose concentrations but was not actively involved in the performance tests to minimize the risk of unblinding remaining investigators and participants to beverage identity since it was expected that CE would increase blood glucose levels.

### Beverage treatments

For the experimental trials, participants received 1 of 3 treatments during the 60-min submaximal exercise bout: water, a grape-flavored 6% carbohydrate-electrolyte (CE) beverage, or a non-caloric grape-flavored beverage containing electrolytes (NCE) and sweetened with sucralose and acesulfame potassium. Beverage treatments were administered to participants in 3 equal aliquots, chilled and in a tinted unmarked bottle at minutes 0, 20, and 40 during the 60-min submaximal cycling bout. Participants were instructed to consume all fluid within a 10–minute period from the time the beverage was received. The mean total beverage volume was 847 ± 368 mL and was equivalent to that participant’s sweat losses based on the familiarization trial.

Study staff and participants were blinded to the caloric and non-caloric beverages but could not be blinded to water. Participants were informed that they would be receiving water and 2 sport beverages during the familiarization session when the purpose of the study was explained, but no other information regarding the beverages was provided. Additionally, participants were instructed not to discuss the characteristics of the beverages with other participants.

### Data analysis

One-way repeated measures analysis of variance was used to analyze differences among beverage trials for WBGT, average HR, peak power for the first W_AnT_, mean power for the first W_AnT_ , mean power averaged across all 3 W_AnT_, S-RPE, and post-exercise questionnaire items. Two-way (beverage × time) repeated measures analysis of variance was used to analyze differences for blood glucose, POMS sub-component scores, and RPE during sub-maximal cycling. Sphericity was confirmed for all comparisons using Mauchly’s test of spehericity. If a significant interaction was found, repeated measures analysis of variance utilizing a Bonferroni-adjusted alpha level was used to analyze simple effects among beverages pre- and post-exercise, and when applicable, differences between individual beverages at specific time points were determined using paired samples t-tests with a Bonferroni-adjusted alpha level. Differences were considered significant if p < 0.05 and data are reported as mean ± SD. All statistical analyses were conducted using PASW version 18.0 (SPSS Inc., Chicago, IL).

Missing data resulted when a pedal came unscrewed during 1 participant’s W_AnT_, 2 individuals did not complete their post-ride evaluation questions after a session, and blood glucose could not be obtained during a single trial for 4 individuals due to a mechanical problem with the analyzer. A series mean method was used to replace these missing data points.

## Results

Environmental conditions were not different among treatments as evidenced by similar WBGT (average across all subjects for all trials = 24.9 ± 0.5°C; Table 2). As intended, exercise intensity, as indexed by average HR (average across all subjects for all trials = 146 ± 4 beats/min) was adequately controlled so participants exercised at similar HR for each trial, as shown in Table 2.

**Table 2:** Characteristics of exercise sessions by treatment

**Variable**	**W**	**NCE**	**CE**
WBGT (°C)	25.0 ± 0.6	25.0 ± 0.5	24.8 ± 0.2
Average HR (beats/min)	145 ± 4	146 ± 4	146 ± 4
Blood Glucose pre-submaximal exercise (mmol/L)	5.6 ± 1.6	5.3 ± 1.6	5.5 ± 1.3
Blood Glucose at end of submaximal exercise (mmol/L)	4.9 ± 1.5†	4.6 ± 1.2^†^	6.1 ± 1.7
POMS Fatigue pre-submaximal exercise‡	1.3 ± 2.0	1.9 ± 2.7	2.0 ± 2.1
POMS Fatigue post-submaximal exercise	4.0 ± 3.3	4.1 ± 2.9	3.4 ± 2.4
POMS Vigor pre-submaximal exercise	6.5 ± 4.7	6.2 ± 4.6	5.8 ± 4.9
POMS Vigor post-submaximal exercise	6.4 ± 5.0	6.5 ± 5.0	6.3 ± 4.8

Data are mean  ±  SD.

†  =  significantly different (p  <  0.05) from CE. ‡  =  beverage by time interaction (p  =  0.04). WBGT  =  wet bulb globe temperature; W  =  water; NCE  =  flavored non-caloric electrolyte beverage; CE  =  flavored caloric electrolyte beverage.

**Figure 1:**
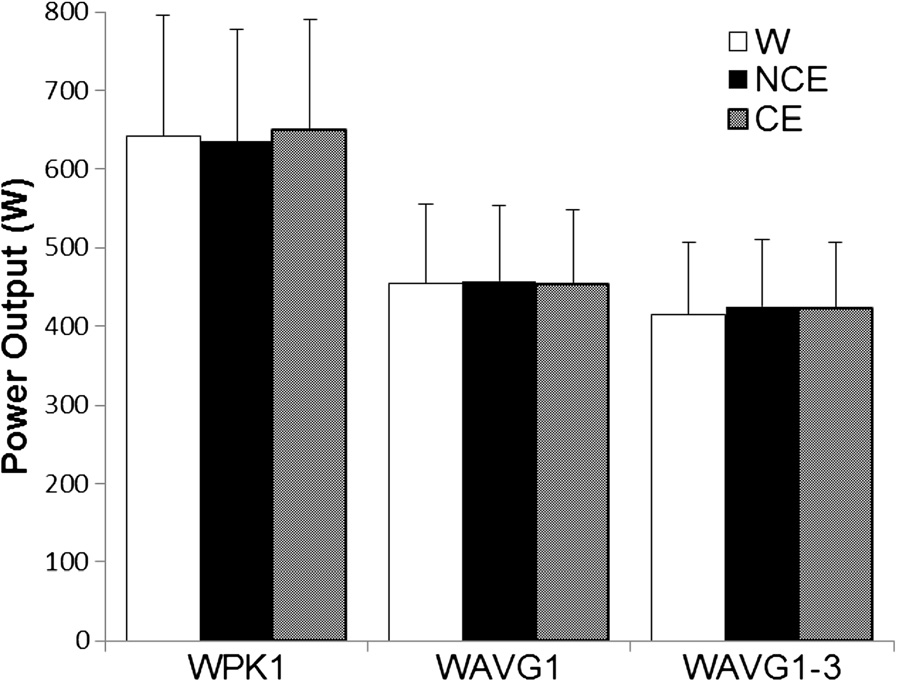
**Wingate Anaerobic Test Performance Outcomes (mean ± SD).** WPK1  =  peak power for the first W_AnT_; WAVG1  =  mean power for the first W_AnT_; WAVG1-3  =  mean power averaged across all 3 W_AnT_; No differences were found among beverages (p  >  0.05). W = water; NCE  =  flavored non-caloric electrolyte beverage; CE  =  flavored caloric electrolyte beverage.

**Figure 2:**
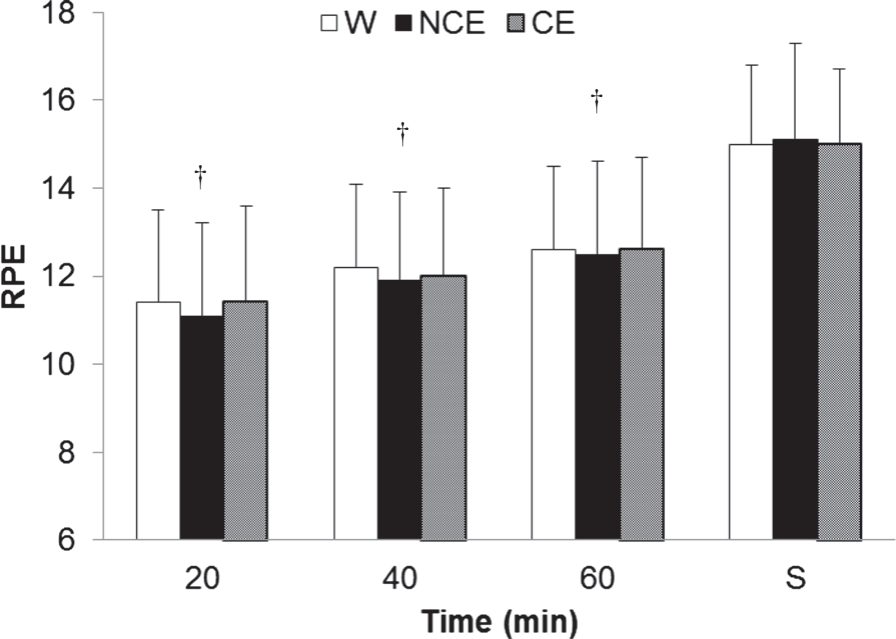
**Ratings of perceived exertion by time point and beverage (mean ± SD).** †  =  (p  <  0.001) between RPE for all other time points during 50 min of sub-maximal cycling. No main effect exhibited for beverage type during sub-maximal cycling (p  =  0.72) or for S (p  =  0.88). S  =  session RPE; W  =  water; NCE  =  flavored non-caloric electrolyte beverage; CE  =  flavored caloric electrolyte beverage.

As expected, blood glucose did not differ among beverages pre-exercise (Table 2), but because of the provision of 49 ± 22 g of carbohydrates in the CE trial, blood glucose was ~ 25% and ~ 32% higher than the W and NCE treatments, respectively, after the 60 min of submaximal exercise (Table 2). Higher blood glucose may have impacted the fatigue rating of the POMS because there was a significant beverage × time interaction (p = 0.04; Table 2). However, no differences were detectable between individual treatments after correcting for experiment-wise alpha level in post hoc multiple comparisons. Nonetheless, the significant interaction means the differences among trials varied based on time, and perhaps this was related to higher blood glucose in the CE trial at the end of exercise, which is consistent with the lower post-exercise fatigue rating in that trial (Table 2). In contrast, provision of exogenous energy via the CE beverage did not affect W_AnT_ performance (Figure 1). There was a main effect (p < 0.001) for time on RPE during sub-maximal cycling, but no effect for beverage during sub-maximal cycling or for S-RPE (average across all subjects for all trials = 15.0 ± 0.3) (Figure 2).

The questionnaire item administered prior to treatment trials revealed that participants did not consume sport beverages on a regular basis (Table 3). Questionnaires completed after exercise during treatment sessions indicated that participants did not believe strongly that consumption of W, NCE, or CE improved performance (Table 3). Beverage treatments did not significantly alter these responses (Table 3). Despite efforts to match target intensity with that which would normally be performed by each participant, they reported exercise difficulty level as more difficult in comparison to their normal workouts, but this outcome was not differently affected by the beverages (Table 3).

**Table 3:** **Responses to 100**-**mm visual analogue scale items**

	**Response Anchors**			
**Item**	**0**	**100**	**Beverage**	**Responses** (**mm**)
1. I regularly drink sport beverages before, during or immediately after exercise.^a^	Never	Always		27.0 ± 28.5
2. Do you feel drinking this beverage during your workout improved your performance ability?^b^	Not at all	Very much	W	45.1 ± 20.4
			NCE	39.7 ± 24.2
			CE	44.7 ± 28.6
3. How difficult was the ride compared to one of your normal workouts?^b^	Much less difficult	Much more difficult	W	60.5 ± 17.1
			NCE	54.9 ± 16.7
			CE	55.6 ± 15.0

Data are mean  ±  SD.

No differences were found among beverages for item 2 and 3 (p > 0.05). W = water; NCE = flavored non-caloric electrolyte beverage; CE  =  flavored caloric electrolyte beverage.

^a^ Item completed during familiarization session after participants described their current physical activity habits.

^b^ Item completed following all exercise during treatment sessions for W, NCE, and CE.

## Discussion

During shorter (~1 h) activity bouts, ingestion of carbohydrates would not be expected to enhance performance because glycogen depletion would be unlikely to limit performance capacity and euglycemia would be maintained without exogenous carbohydrate ingestion [8,11]. However, numerous investigations typically involving highly trained endurance athletes running or cycling after periods of significant fasting have provided evidence supporting enhanced performance and mood or lowered perceived exertion during exercise lasting ~1 h with CE ingestion or mouth rinse, without confirmation of the mechanisms responsible for these changes. The aims of this study were to determine if similar ergogenic properties would be exhibited in non-fasted recreational exercisers.

The results of this study support our first hypothesis that CE consumption during 50 min of sub-maximal exercise would not result in improved W_AnT_ performance compared to NCE or W (Figure 1). Ball et al. [5] found carbohydrate ingestion during 50 min of high intensity cycling resulted in 6.5% higher mean power and 5.8% higher peak power during a subsequent W_AnT_ versus ingesting an artificially-sweetened placebo. The similarity in protocols makes comparing the results between the current and Ball et al. [5] studies favorable with 3 factors taken into consideration.

The first is that the 50 min sub-maximal exercise intensity was prescribed at a more moderate intensity level that could be completed by our highly active but non-competitive level recreational exercisers. It is possible that our contrasting finding of no impact of carbohydrate consumption on performance was due to the lower relative intensity level of the sub-maximal exercise portion of our protocol, which resulted in 15 beats per min lower mean HR than was exhibited for the participants in the Ball et al. [5] study. However, mean sub-maximal exercise RPEs in the Ball et al. [5] study were only 5.0 ± 1.0 (carbohydrate trial) and 5.6 ± 1.1(placebo trial), and our participants reported the overall difficulty of the trials was higher than their normal workouts (Table 3).

A second difference in our methodology and that of Ball et al. [5] was that our protocol incorporated 3 sets of W_AnT_ versus a single W_AnT_ to assess performance. The primary rationale for incorporating W_AnT_ as a performance measure was that variability in pacing strategies for our recreational exercisers would make it difficult to interpret more aerobically-based time trial tests that have been most commonly used to assess performance differences in the past. However, repeated W_AnT_ have been established to be a stable measure, particularly if a practice session is provided [33] and allowed for direct comparison to the results of the Ball et al. [5] study. The additional two W_AnT_ were used to ensure fatigue late in exercise, as we anticipated our sub-maximal exercise bout would be comparatively less intense based on average heart rate than that of Ball et al. [5]. We predicted if CE ingestion exhibited a protective effect and delayed fatigue, the effects would be more likely to be manifested with repeated versus a single W_AnT_. Carbohydrate ingestion during ~1 h of intermittent high intensity exercise has also been shown to improve multiple forms of anaerobic performance tests late in exercise including 20–m sprint time [12,13], vertical jump height [13], and shuttle running to fatigue [12] for recreational athletes.

A third consideration when comparing our findings was that of the competitive cyclists in Ball et al. [5] were that Ball et al.’s participants fasted for 12 h prior to exercise. In contrast, in the present study and others [21-25] a pre-activity meal was consumed within 2 to 4 hours before the start of exercise. All of the studies that included pre-activity meals found no increase in performance with carbohydrate consumption or mouth rinse during activity. Pre-feeding provides contrasting results (i.e. no improvement versus improvement) compared to nearly all published investigations incorporating fasted participants in exercise lasting 1 h or less. The findings of the present study using recreational exercisers supports the position of Desbrow et al. [21] who studied highly trained cyclists, and found that mixed-nutrient feeding within a few hours prior to testing mitigated most ergogenic effects of carbohydrate ingestion during exercise of ~1 hour in duration. As long as gastrointestinal distress is not a concern, a pre-exercise meal is recommended for athletes, and beginning exercise in a fasted state is discouraged [34]. In light of our findings and those of others who included a pre-activity meal in their study design, as well as in keeping with the recommendations for athletes by most sport nutrition related organizations [34], the impact of including a meal or snack in a reasonable time frame prior to exercise warrants further discussion.

In addition to performance improvement, Ball et al. [5] found significantly lower mean RPEs for competitive cyclists consuming a CE versus a placebo. Although blood glucose was not measured in their investigation, the authors speculated the differences in RPE for their cyclists possibly stemmed from higher levels of blood glucose maintenance with carbohydrate ingestion versus placebo [5]. In our investigation, CE resulted in higher blood glucose levels at the end of sub-maximal cycling, but normal blood glucose levels were observed for NCE or W treatments. Sweetness, whether from caloric or non-caloric sources, did not result in statistical differences in perceived exertion (Figure 2) or POMS responses (Table 2) in comparison to each other or W.

Authors of other studies have suggested that improved mood and lower perceived exertion associated with carbohydrate ingestion or mouth rinse may be mediated through central neural mechanisms [5,12,13,15,19]. Functional magnetic resonance imaging has revealed that sucrose, delivered in small doses of fluid to the mouth, results in reward center activation in the brain that is not exhibited with the artificial sweetener sucralose [35]. Similar results have also been found for other forms of less sweet carbohydrate sources such as maltodextrin and glucose compared to saccharin [14]. Artificial sweeteners do not elicit the same response as carbohydrates whether participants are fed [35] or fasted [14]. Obvious technical limitations of functional MRI make it difficult to determine if physical activity alters these responses, but under the exercise conditions of the present investigation, the addition of caloric sweeteners do not appear to provide an affective domain advantage. If these unidentified oral receptors are responsible for lessened perception of fatigue, it is plausible that their impact is mitigated by carbohydrate presence in the gastrointestinal tract, or changes in blood glucose or glycogen concentration levels in liver or muscle tissue following a pre-exercise meal.

Perhaps part of the reason the mood of our participants was not affected by the CE treatment is because our participants had preconceived notions regarding the efficacy of sport beverages (Table 3). While regularly physically active, our participants were neither competitive nor elite endurance athletes, who have been shown to have strong convictions that CE can improve performance [36,37]. In one study, following a 40-km time trial with water ingestion only, competitive cyclists were split into 2 cohorts with 1 group being told they were going to consume a CE and the other group being told they were receiving a carbohydrate-free artificially sweetened beverage. In actuality, half of the cyclists in each group received a placebo, and the other half received a CE. The group informed that they were receiving CE improved their average power output by 4.3% during a second time trial compared to baseline whereas the group informed that they were receiving a carbohydrate-free artificially flavored beverage increased their power output by only 0.5%, even though half of the individuals in both groups actually received a CE [36].

Differences between the participants in the present study and competitive endurance athletes featured in other studies [36,37] may be related to exposure of competitive athletes to literature promoting the importance of CE for performance. It is also probable that most participants in the current investigation were unlikely to have had experiences in which they felt a lack of exogenous carbohydrates hindered exercise performance in comparison to the competitive endurance athletes used in other investigations. These factors may have given our participants a different subjective bias concerning mood and perceived exertion, in contrast to those of trained endurance athletes who frequently consume CE.

## Conclusions

The findings of this study suggest recreational exercisers should expect no decrease in performance capacity in exercise lasting ~1 h and should not feel more fatigued or perceive exercise as more difficult when W or a NCE is substituted for a CE, under conditions similar to those of the present study. These results further strengthen the position from similar studies investigating CE effects on running or cycling performances lasting ~1 h that no ergogenic effects are exhibited when subjects consume a pre-activity meal. The discrepant findings from studies with fasted athletes highlights the impact pre-exercise feeding protocols may have on the results of sport beverage studies and should be given consideration in future CE study design.

## Competing interests

Equipment and beverages used in this investigation were prepared and provided by The Coca-Cola Co. Financial compensation was also awarded to the subjects for their participation and investigators EO and PB for designing, directing, collecting data and writing this manuscript. SP is employed by The Coca-Cola Co.

## Authors’ contributions

EKO developed the study design, collected data, conducted statistical analysis, and drafted and submitted the manuscript. PAB, SPP, JEW, and MTR assisted in the study design, interpretation of data, and critically reviewed the manuscript. All authors read and approved the final manuscript.
